# Poly[di-μ_2_-acetato-diaquabis(2,2′-bi­pyridine)bis(μ_3_-5-nitroisophthalato)tricobalt(II)]

**DOI:** 10.1107/S1600536809012562

**Published:** 2009-04-10

**Authors:** Hai-Dong Wang, Min-Min Li, Hong-Yin He

**Affiliations:** a Biological and Chemical Engineering School of Jiaxing University, Jiaxing 314001, People’s Republic of China

## Abstract

The title complex, [Co_3_(C_8_H_3_NO_6_)_2_(C_2_H_3_O_2_)_2_(C_10_H_8_N_2_)_2_(H_2_O)_2_], was synthesized under hydro­thermal conditions. The structure features a centrosymmetric complex with three Co^II^ centres, one of which is located on a centre of inversion. The Co centres are coordinated in a distorted octa­hedral geometry. The bipyridine ligands are bonded to just one Co centre in a chelating mode, whereas the 5-nitro­isophthalate and acetate ions are bonded to two different Co atoms. The crystal structure is stabilized by O—H⋯O hydrogen bonds.

## Related literature

For related structures, see: He *et al.* (2004 ▶, 2005 ▶); Zhang *et al.* (2006 ▶).
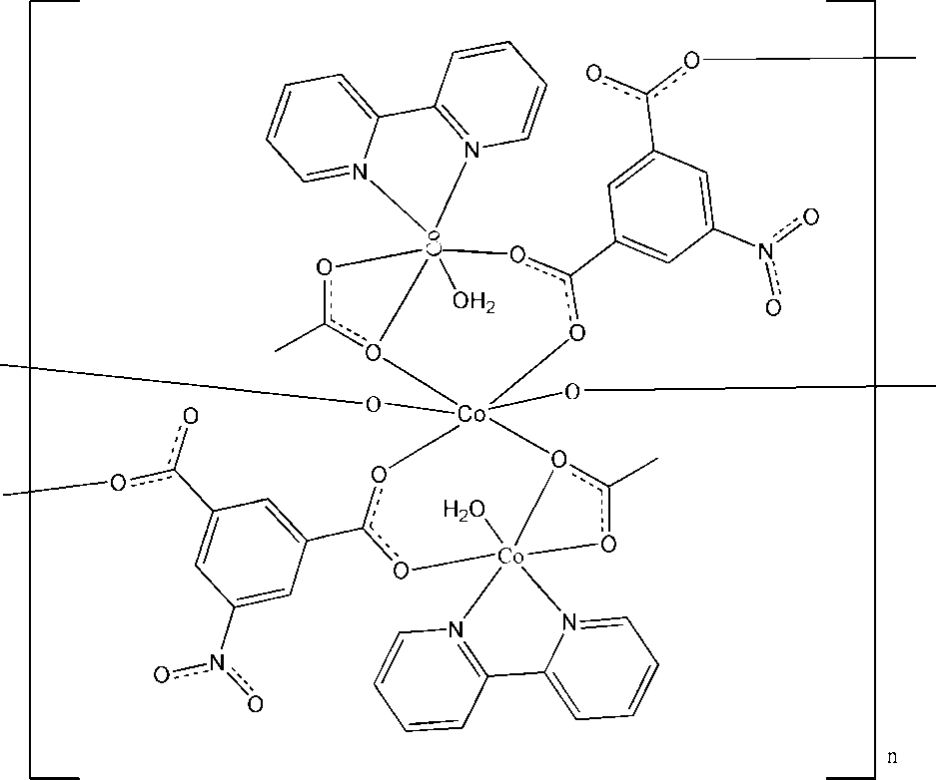


## Experimental

### 

#### Crystal data

[Co_3_(C_8_H_3_NO_6_)_2_(C_2_H_3_O_2_)_2_(C_10_H_8_N_2_)_2_(H_2_O)_2_]
*M* *_r_* = 1061.51
Triclinic, 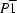

*a* = 10.0084 (1) Å
*b* = 10.0781 (1) Å
*c* = 11.3941 (1) Å
α = 81.196 (1)°
β = 67.685 (1)°
γ = 69.472 (1)°
*V* = 995.43 (2) Å^3^
*Z* = 1
Mo *K*α radiation
μ = 1.33 mm^−1^
*T* = 296 K
0.26 × 0.13 × 0.10 mm

#### Data collection

Bruker SMART 1K CCD diffractometer
Absorption correction: multi-scan (*SADABS*; Bruker, 2002 ▶) *T*_min_ = 0.724, *T*_max_ = 0.883
10424 measured reflections
3679 independent reflections
3296 reflections with *I* > 2σ(*I*)
*R*_int_ = 0.020

#### Refinement

*R*[*F* ^2^ > 2σ(*F* ^2^)] = 0.030
*wR*(*F* ^2^) = 0.077
*S* = 1.03
3679 reflections
310 parameters
3 restraints
H atoms treated by a mixture of independent and constrained refinement
Δρ_max_ = 0.53 e Å^−3^
Δρ_min_ = −0.45 e Å^−3^

### 

Data collection: *SMART* (Bruker, 2002 ▶); cell refinement: *SAINT* (Bruker, 2002 ▶); data reduction: *SAINT*; program(s) used to solve structure: *SHELXS97* (Sheldrick, 2008 ▶); program(s) used to refine structure: *SHELXL97* (Sheldrick, 2008 ▶); molecular graphics: *ORTEP-3 for Windows* (Farrugia, 1997 ▶); software used to prepare material for publication: *PLATON* (Spek, 2009 ▶).

## Supplementary Material

Crystal structure: contains datablocks I, added_by_encifer. DOI: 10.1107/S1600536809012562/bt2923sup1.cif

Structure factors: contains datablocks I. DOI: 10.1107/S1600536809012562/bt2923Isup2.hkl

Additional supplementary materials:  crystallographic information; 3D view; checkCIF report

## Figures and Tables

**Table 1:** Hydrogen-bond geometry (Å, °)

*D*—H⋯*A*	*D*—H	H⋯*A*	*D*⋯*A*	*D*—H⋯*A*
O1*W*—H1*WA*⋯O3^i^	0.83 (2)	1.99 (2)	2.755 (3)	154 (2)
O1*W*—H1*WB*⋯O3^ii^	0.826 (18)	1.96 (2)	2.762 (3)	163 (3)

Symmetry codes: (i) 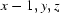; (ii) 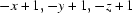.

**Fig. 1.**
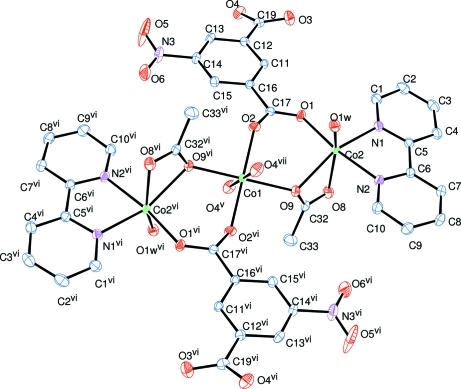
View of the title complex view. Displacement ellipsoids are drawn at the 30% probability level. H atoms are omitted for clarity. Symmetry codes for generating equivalent atoms: (v) x-1, y, z. (vi) x, -y, 1-z. (vii) 1-x, 2-y, 1-z.

## supplementary crystallographic information

### Comment

Recently, we were interested in polymers formed by isophthalate ligands and the complexes the form with transition metals  because of their diverse topologies and potential applications as functional materials (He *et al.*,  2004, 2005, Zhang *et al.*, 2006).

The structure features a centrosymmetric complex with three  Co(II) centres, one of which is located on a centre of inversion. The Co centres are coordinated in a distorted octahedral  geometry. The bipyridine ligands are bonded to just one Co centre in a chelating mode, whereas the 5-nitroisophthalate and acetate ions are bonded to two different Co atoms. The crystal structure is stabilized by O-H···O hydrogen bonds (Tab. 1).

### Experimental

A mixture of Co(Ac)_2_.4H_2_O (0.1240g, 0.5 mmol), 2,2'-bipyridine (0.0790g, 0.5 mmol), 5-nitroisophthalic acid (0.1050g, 0.5mmol), 8 ml H_2_O and 8ml EtOH was heated at 413 K for three days in a 20 ml Teflon-lined stainless-steel autoclave. After cooling, a red plate shaped crystals of the title compound were obstained.

### Refinement

The H atoms of aromatic and methyl group were positioned geometrically, and included in the refinement in the riding model approximation with C-H = 0.93 Å for aromatic H atoms and C-H = 0.96 Å for H atoms of methyl groups and U_iso_=1.2Ueq(C). The H atoms of the water molecule were found in a difference Fourier map and refined isotropically with the O-H bonds restrained to 0.82 (1)Å and the H···H distance restrained to 1.4 (1)Å.

### Crystal data

**Table:**

[Co_3_(C_8_H_3_NO_6_)_2_(C_2_H_3_O_2_)_2_(C_10_H_8_N_2_)_2_(H_2_O)_2_]	*Z* = 1
*M**_r_* = 1061.51	*F*(000) = 539
Triclinic, *P*1	*D*_x_ = 1.771 Mg m^−^^3^
*a* = 10.0084 (1) Å	Mo *K*α radiation, λ = 0.71073 Å
*b* = 10.0781 (1) Å	Cell parameters from 10424 reflections
*c* = 11.3941 (1) Å	θ = 1.9–25.5°
α = 81.196 (1)°	µ = 1.33 mm^−^^1^
β = 67.685 (1)°	*T* = 296 K
γ = 69.472 (1)°	Plate, red
*V* = 995.43 (2) Å^3^	0.26 × 0.13 × 0.10 mm

### Data collection

**Table:**

Bruker SMART 1K CCD diffractometer	3679 independent reflections
Radiation source: fine-focus sealed tube	3296 reflections with *I* > 2σ(*I*)
graphite	*R*_int_ = 0.020
phi/ω scans	θ_max_ = 25.5°, θ_min_ = 1.9°
Absorption correction: multi-scan (*SADABS*; Bruker, 2002)	*h* = −12→12
*T*_min_ = 0.724, *T*_max_ = 0.883	*k* = −12→12
10424 measured reflections	*l* = −13→13

### Refinement

**Table:**

Refinement on *F*^2^	Primary atom site location: structure-invariant direct methods
Least-squares matrix: full	Secondary atom site location: difference Fourier map
*R*[*F*^2^ > 2σ(*F*^2^)] = 0.030	Hydrogen site location: inferred from neighbouring sites
*wR*(*F*^2^) = 0.077	H atoms treated by a mixture of independent and constrained refinement
*S* = 1.03	*w* = 1/[σ^2^(*F*_o_^2^) + (0.0358*P*)^2^ + 1.058*P*] where *P* = (*F*_o_^2^ + 2*F*_c_^2^)/3
3679 reflections	(Δ/σ)_max_ < 0.001
310 parameters	Δρ_max_ = 0.53 e Å^−^^3^
3 restraints	Δρ_min_ = −0.45 e Å^−^^3^

### Special details

**Table:**

Geometry. All esds (except the esd in the dihedral angle between two l.s. planes) are estimated using the full covariance matrix. The cell esds are taken into account individually in the estimation of esds in distances, angles and torsion angles; correlations between esds in cell parameters are only used when they are defined by crystal symmetry. An approximate (isotropic) treatment of cell esds is used for estimating esds involving l.s. planes.
Refinement. Refinement of F^2^ against ALL reflections. The weighted R-factor wR and goodness of fit S are based on F^2^, conventional R-factors R are based on F, with F set to zero for negative F^2^. The threshold expression of F^2^ > 2sigma(F^2^) is used only for calculating R-factors(gt) etc. and is not relevant to the choice of reflections for refinement. R-factors based on F^2^ are statistically about twice as large as those based on F, and R- factors based on ALL data will be even larger.

### Fractional atomic coordinates and isotropic or equivalent isotropic displacement parameters (Å^2^)

**Table:**

	*x*	*y*	*z*	*U*_iso_*/*U*_eq_	
Co1	0.0000	1.0000	0.5000	0.02056 (11)	
Co2	0.29863 (3)	0.66172 (3)	0.34001 (3)	0.02348 (10)	
N1	0.5014 (2)	0.4947 (2)	0.26205 (19)	0.0264 (4)	
N2	0.2395 (2)	0.5533 (2)	0.23098 (19)	0.0271 (4)	
C6	0.3533 (3)	0.4463 (2)	0.1610 (2)	0.0259 (5)	
O1	0.38924 (18)	0.74188 (17)	0.43457 (16)	0.0283 (4)	
O9	0.10814 (18)	0.86327 (17)	0.34394 (15)	0.0263 (4)	
C11	0.5544 (3)	0.8074 (2)	0.5603 (2)	0.0218 (5)	
H11A	0.6088	0.7353	0.5017	0.026*	
C17	0.3124 (3)	0.8272 (2)	0.5243 (2)	0.0233 (5)	
C16	0.3980 (3)	0.8686 (2)	0.5891 (2)	0.0219 (5)	
C19	0.8024 (3)	0.7950 (3)	0.5801 (2)	0.0269 (5)	
C12	0.6310 (3)	0.8526 (2)	0.6180 (2)	0.0233 (5)	
O3	0.8695 (2)	0.67757 (19)	0.5289 (2)	0.0414 (5)	
C5	0.5033 (3)	0.4180 (2)	0.1738 (2)	0.0256 (5)	
C4	0.6363 (3)	0.3199 (3)	0.1018 (2)	0.0337 (6)	
H4A	0.6361	0.2703	0.0395	0.040*	
C14	0.3935 (3)	1.0141 (2)	0.7364 (2)	0.0273 (5)	
C13	0.5491 (3)	0.9578 (3)	0.7079 (2)	0.0272 (5)	
H13A	0.5977	0.9894	0.7478	0.033*	
C15	0.3160 (3)	0.9740 (2)	0.6786 (2)	0.0262 (5)	
H15A	0.2114	1.0163	0.6989	0.031*	
C9	0.0689 (3)	0.5133 (3)	0.1519 (3)	0.0382 (6)	
H9A	−0.0288	0.5389	0.1495	0.046*	
C7	0.3293 (3)	0.3687 (3)	0.0849 (3)	0.0352 (6)	
H7A	0.4092	0.2951	0.0369	0.042*	
C10	0.1008 (3)	0.5858 (3)	0.2260 (3)	0.0328 (6)	
H10A	0.0225	0.6601	0.2742	0.039*	
C1	0.6309 (3)	0.4698 (3)	0.2832 (3)	0.0327 (6)	
H1A	0.6295	0.5217	0.3447	0.039*	
C8	0.1844 (3)	0.4028 (3)	0.0818 (3)	0.0406 (7)	
H8A	0.1652	0.3512	0.0324	0.049*	
C2	0.7661 (3)	0.3707 (3)	0.2180 (3)	0.0379 (6)	
H2A	0.8533	0.3539	0.2369	0.046*	
N3	0.3069 (3)	1.1250 (3)	0.8321 (2)	0.0462 (6)	
C3	0.7690 (3)	0.2970 (3)	0.1241 (3)	0.0392 (6)	
H3A	0.8598	0.2323	0.0761	0.047*	
O6	0.1710 (3)	1.1769 (3)	0.8577 (3)	0.0776 (9)	
O2	0.17072 (18)	0.88101 (19)	0.56665 (16)	0.0318 (4)	
O4	0.8652 (2)	0.8704 (2)	0.60355 (17)	0.0369 (4)	
O5	0.3732 (3)	1.1514 (4)	0.8890 (3)	0.1161 (15)	
O1W	0.17349 (19)	0.56964 (19)	0.50084 (18)	0.0318 (4)	
H1WA	0.0831 (15)	0.620 (2)	0.523 (3)	0.048*	
H1WB	0.180 (3)	0.4895 (15)	0.486 (3)	0.048*	
O8	0.31763 (19)	0.80788 (18)	0.17845 (16)	0.0334 (4)	
C32	0.1871 (3)	0.8911 (2)	0.2305 (2)	0.0263 (5)	
C33	0.1213 (4)	1.0190 (3)	0.1602 (3)	0.0468 (7)	
H33A	0.1949	1.0239	0.0772	0.070*	
H33B	0.0952	1.1025	0.2056	0.070*	
H33C	0.0314	1.0129	0.1524	0.070*	

### Atomic displacement parameters (Å^2^)

**Table:**

	*U*^11^	*U*^22^	*U*^33^	*U*^12^	*U*^13^	*U*^23^
Co1	0.0155 (2)	0.0220 (2)	0.0263 (2)	−0.00532 (16)	−0.00810 (17)	−0.00619 (17)
Co2	0.02330 (17)	0.02240 (17)	0.02775 (18)	−0.00702 (13)	−0.01024 (13)	−0.00655 (13)
N1	0.0258 (10)	0.0226 (10)	0.0315 (11)	−0.0068 (8)	−0.0108 (8)	−0.0030 (8)
N2	0.0270 (10)	0.0274 (10)	0.0295 (11)	−0.0090 (8)	−0.0106 (9)	−0.0057 (8)
C6	0.0327 (13)	0.0215 (11)	0.0249 (12)	−0.0087 (10)	−0.0114 (10)	−0.0010 (9)
O1	0.0230 (8)	0.0311 (9)	0.0350 (9)	−0.0075 (7)	−0.0115 (7)	−0.0123 (7)
O9	0.0279 (9)	0.0240 (8)	0.0270 (9)	−0.0078 (7)	−0.0085 (7)	−0.0052 (7)
C11	0.0251 (11)	0.0195 (11)	0.0236 (11)	−0.0089 (9)	−0.0092 (9)	−0.0022 (9)
C17	0.0251 (12)	0.0214 (11)	0.0255 (12)	−0.0074 (9)	−0.0121 (9)	0.0016 (9)
C16	0.0243 (11)	0.0212 (11)	0.0221 (11)	−0.0085 (9)	−0.0093 (9)	−0.0001 (9)
C19	0.0258 (12)	0.0310 (13)	0.0279 (12)	−0.0145 (10)	−0.0099 (10)	0.0027 (10)
C12	0.0256 (12)	0.0219 (11)	0.0256 (11)	−0.0115 (9)	−0.0099 (9)	0.0020 (9)
O3	0.0261 (9)	0.0334 (10)	0.0636 (13)	−0.0083 (8)	−0.0114 (9)	−0.0124 (9)
C5	0.0301 (12)	0.0213 (11)	0.0248 (12)	−0.0083 (10)	−0.0093 (10)	0.0005 (9)
C4	0.0369 (14)	0.0273 (13)	0.0303 (13)	−0.0046 (11)	−0.0087 (11)	−0.0040 (10)
C14	0.0327 (13)	0.0255 (12)	0.0241 (12)	−0.0113 (10)	−0.0063 (10)	−0.0070 (10)
C13	0.0337 (13)	0.0317 (13)	0.0246 (12)	−0.0182 (11)	−0.0113 (10)	−0.0029 (10)
C15	0.0239 (12)	0.0250 (12)	0.0274 (12)	−0.0058 (9)	−0.0073 (9)	−0.0040 (10)
C9	0.0380 (15)	0.0400 (15)	0.0478 (16)	−0.0155 (12)	−0.0235 (13)	−0.0031 (13)
C7	0.0434 (15)	0.0287 (13)	0.0365 (14)	−0.0074 (11)	−0.0180 (12)	−0.0090 (11)
C10	0.0288 (13)	0.0325 (13)	0.0387 (14)	−0.0082 (11)	−0.0117 (11)	−0.0098 (11)
C1	0.0318 (13)	0.0284 (13)	0.0433 (15)	−0.0104 (11)	−0.0180 (11)	−0.0015 (11)
C8	0.0552 (18)	0.0359 (15)	0.0451 (16)	−0.0165 (13)	−0.0280 (14)	−0.0090 (12)
C2	0.0274 (13)	0.0329 (14)	0.0530 (17)	−0.0102 (11)	−0.0160 (12)	0.0063 (13)
N3	0.0444 (15)	0.0481 (14)	0.0441 (14)	−0.0132 (12)	−0.0057 (11)	−0.0256 (12)
C3	0.0300 (14)	0.0309 (14)	0.0419 (15)	−0.0007 (11)	−0.0053 (12)	−0.0006 (12)
O6	0.0595 (16)	0.0778 (18)	0.0809 (18)	0.0265 (13)	−0.0342 (14)	−0.0527 (15)
O2	0.0200 (8)	0.0421 (10)	0.0341 (9)	−0.0035 (7)	−0.0137 (7)	−0.0073 (8)
O4	0.0339 (10)	0.0497 (11)	0.0392 (10)	−0.0286 (9)	−0.0109 (8)	−0.0037 (9)
O5	0.0539 (16)	0.176 (3)	0.130 (3)	−0.0378 (19)	0.0024 (16)	−0.128 (3)
O1W	0.0276 (9)	0.0288 (9)	0.0382 (10)	−0.0091 (7)	−0.0093 (8)	−0.0044 (8)
O8	0.0297 (9)	0.0333 (9)	0.0322 (9)	−0.0078 (8)	−0.0055 (8)	−0.0059 (8)
C32	0.0309 (13)	0.0255 (12)	0.0277 (12)	−0.0107 (10)	−0.0125 (10)	−0.0058 (10)
C33	0.0542 (18)	0.0416 (16)	0.0379 (16)	−0.0079 (14)	−0.0182 (14)	0.0054 (13)

### Geometric parameters (Å, °)

**Table:**

Co1—O2	2.0503 (16)	C5—C4	1.384 (3)
Co1—O2^i^	2.0503 (16)	C4—C3	1.381 (4)
Co1—O9^i^	2.1153 (15)	C4—H4A	0.9300
Co1—O9	2.1153 (15)	C14—C15	1.375 (3)
Co1—O4^ii^	2.1154 (17)	C14—C13	1.380 (4)
Co1—O4^iii^	2.1154 (17)	C14—N3	1.471 (3)
Co2—O1	2.0392 (15)	C13—H13A	0.9300
Co2—O1W	2.0831 (18)	C15—H15A	0.9300
Co2—N1	2.1053 (19)	C9—C8	1.372 (4)
Co2—N2	2.1249 (19)	C9—C10	1.380 (4)
Co2—O8	2.1668 (18)	C9—H9A	0.9300
Co2—O9	2.2382 (16)	C7—C8	1.382 (4)
N1—C1	1.338 (3)	C7—H7A	0.9300
N1—C5	1.350 (3)	C10—H10A	0.9300
N2—C10	1.331 (3)	C1—C2	1.377 (4)
N2—C6	1.345 (3)	C1—H1A	0.9300
C6—C7	1.385 (3)	C8—H8A	0.9300
C6—C5	1.487 (3)	C2—C3	1.380 (4)
O1—C17	1.259 (3)	C2—H2A	0.9300
O9—C32	1.281 (3)	N3—O5	1.199 (3)
C11—C16	1.391 (3)	N3—O6	1.206 (3)
C11—C12	1.395 (3)	C3—H3A	0.9300
C11—H11A	0.9300	O4—Co1^iv^	2.1154 (17)
C17—O2	1.248 (3)	O1W—H1WA	0.826 (10)
C17—C16	1.508 (3)	O1W—H1WB	0.824 (10)
C16—C15	1.388 (3)	O8—C32	1.248 (3)
C19—O3	1.244 (3)	C32—C33	1.493 (4)
C19—O4	1.252 (3)	C33—H33A	0.9600
C19—C12	1.510 (3)	C33—H33B	0.9600
C12—C13	1.390 (3)	C33—H33C	0.9600
			
O2—Co1—O2^i^	180.0	C13—C12—C19	119.0 (2)
O2—Co1—O9^i^	92.58 (6)	C11—C12—C19	121.4 (2)
O2^i^—Co1—O9^i^	87.42 (6)	N1—C5—C4	121.7 (2)
O2—Co1—O9	87.42 (6)	N1—C5—C6	115.1 (2)
O2^i^—Co1—O9	92.58 (6)	C4—C5—C6	123.2 (2)
O9^i^—Co1—O9	180.000 (1)	C3—C4—C5	119.0 (2)
O2—Co1—O4^ii^	90.67 (7)	C3—C4—H4A	120.5
O2^i^—Co1—O4^ii^	89.33 (7)	C5—C4—H4A	120.5
O9^i^—Co1—O4^ii^	88.72 (7)	C15—C14—C13	123.5 (2)
O9—Co1—O4^ii^	91.28 (7)	C15—C14—N3	118.4 (2)
O2—Co1—O4^iii^	89.33 (7)	C13—C14—N3	118.1 (2)
O2^i^—Co1—O4^iii^	90.67 (7)	C14—C13—C12	118.1 (2)
O9^i^—Co1—O4^iii^	91.28 (7)	C14—C13—H13A	120.9
O9—Co1—O4^iii^	88.72 (7)	C12—C13—H13A	120.9
O4^ii^—Co1—O4^iii^	180.0	C14—C15—C16	118.3 (2)
O1—Co2—O1W	94.98 (7)	C14—C15—H15A	120.9
O1—Co2—N1	94.01 (7)	C16—C15—H15A	120.9
O1W—Co2—N1	103.72 (7)	C8—C9—C10	118.6 (2)
O1—Co2—N2	170.78 (7)	C8—C9—H9A	120.7
O1W—Co2—N2	87.41 (7)	C10—C9—H9A	120.7
N1—Co2—N2	76.77 (7)	C8—C7—C6	118.7 (2)
O1—Co2—O8	98.32 (7)	C8—C7—H7A	120.7
O1W—Co2—O8	152.53 (7)	C6—C7—H7A	120.7
N1—Co2—O8	99.22 (7)	N2—C10—C9	122.5 (2)
N2—Co2—O8	83.29 (7)	N2—C10—H10A	118.8
O1—Co2—O9	94.50 (6)	C9—C10—H10A	118.8
O1W—Co2—O9	95.80 (7)	N1—C1—C2	123.0 (2)
N1—Co2—O9	157.91 (7)	N1—C1—H1A	118.5
N2—Co2—O9	94.12 (7)	C2—C1—H1A	118.5
O8—Co2—O9	59.37 (6)	C9—C8—C7	119.6 (2)
C1—N1—C5	118.4 (2)	C9—C8—H8A	120.2
C1—N1—Co2	124.99 (16)	C7—C8—H8A	120.2
C5—N1—Co2	116.21 (15)	C1—C2—C3	118.4 (2)
C10—N2—C6	119.0 (2)	C1—C2—H2A	120.8
C10—N2—Co2	124.78 (16)	C3—C2—H2A	120.8
C6—N2—Co2	116.22 (15)	O5—N3—O6	122.5 (3)
N2—C6—C7	121.6 (2)	O5—N3—C14	117.9 (3)
N2—C6—C5	114.8 (2)	O6—N3—C14	119.3 (2)
C7—C6—C5	123.6 (2)	C2—C3—C4	119.5 (2)
C17—O1—Co2	124.66 (14)	C2—C3—H3A	120.3
C32—O9—Co1	126.76 (14)	C4—C3—H3A	120.3
C32—O9—Co2	88.61 (13)	C17—O2—Co1	138.15 (16)
Co1—O9—Co2	121.97 (8)	C19—O4—Co1^iv^	137.23 (17)
C16—C11—C12	121.0 (2)	Co2—O1W—H1WA	109 (2)
C16—C11—H11A	119.5	Co2—O1W—H1WB	111 (2)
C12—C11—H11A	119.5	H1WA—O1W—H1WB	109 (2)
O2—C17—O1	126.4 (2)	C32—O8—Co2	92.74 (15)
O2—C17—C16	116.0 (2)	O8—C32—O9	119.2 (2)
O1—C17—C16	117.6 (2)	O8—C32—C33	120.5 (2)
C15—C16—C11	119.6 (2)	O9—C32—C33	120.2 (2)
C15—C16—C17	117.9 (2)	C32—C33—H33A	109.5
C11—C16—C17	122.5 (2)	C32—C33—H33B	109.5
O3—C19—O4	125.4 (2)	H33A—C33—H33B	109.5
O3—C19—C12	118.0 (2)	C32—C33—H33C	109.5
O4—C19—C12	116.6 (2)	H33A—C33—H33C	109.5
C13—C12—C11	119.5 (2)	H33B—C33—H33C	109.5

Symmetry codes: (i) −*x*, −*y*+2, −*z*+1; (ii) *x*−1, *y*, *z*; (iii) −*x*+1, −*y*+2, −*z*+1; (iv) *x*+1, *y*, *z*.

### Hydrogen-bond geometry (Å, °)

**Table:**

*D*—H···*A*	*D*—H	H···*A*	*D*···*A*	*D*—H···*A*
O1W—H1WA···O3^ii^	0.83 (2)	1.99 (2)	2.755 (3)	154 (2)
O1W—H1WB···O3^v^	0.83 (2)	1.96 (2)	2.762 (3)	163 (3)

Symmetry codes: (ii) *x*−1, *y*, *z*; (v) −*x*+1, −*y*+1, −*z*+1.
